# Focal intra-colon cooling reduces organ injury and systemic inflammation after REBOA management of lethal hemorrhage in rats

**DOI:** 10.1038/s41598-021-93064-4

**Published:** 2021-07-01

**Authors:** Awadhesh K. Arya, Kurt Hu, Lalita Subedi, Tieluo Li, Bingren Hu

**Affiliations:** 1 Departments of Anesthesiology, Shock, Trauma and Anesthesiology Research Center, University of Maryland School of Medicine, Baltimore, MD USA; 2 Department of Medicine, Division of Pulmonary and Critical Care Medicine, Medical College of Wisconsin, Milwaukee, WI USA; 3 Veterans Affairs Maryland Health Center System, 10 North Greene Street, Baltimore, MD USA

**Keywords:** Biological techniques, Biomarkers, Diseases, Medical research, Pathogenesis

## Abstract

Resuscitative endovascular balloon occlusion of the aorta (REBOA) is a lifesaving maneuver for the management of lethal torso hemorrhage. However, its prolonged use leads to distal organ ischemia–reperfusion injury (IRI) and systemic inflammatory response syndrome (SIRS). The objective of this study is to investigate the blood-based biomarkers of IRI and SIRS and the efficacy of direct intestinal cooling in the prevention of IRI and SIRS. A rat lethal hemorrhage model was produced by bleeding 50% of the total blood volume. A balloon catheter was inserted into the aorta for the implementation of REBOA. A novel TransRectal Intra-Colon (TRIC) device was placed in the descending colon and activated from 10 min after the bleeding to maintain the intra-colon temperature at 37 °C (TRIC37°C group) or 12 °C (TRIC12°C group) for 270 min. The upper body temperature was maintained at as close to 37 °C as possible in both groups. Blood samples were collected before hemorrhage and after REBOA. The organ injury biomarkers and inflammatory cytokines were evaluated by ELISA method. Blood based organ injury biomarkers (endotoxin, creatinine, AST, FABP1/L-FABP, cardiac troponin I, and FABP2/I-FABP) were all drastically increased in TRIC37°C group after REBOA. TRIC12°C significantly downregulated these increased organ injury biomarkers. Plasma levels of pro-inflammatory cytokines TNF-α, IL-1b, and IL-17F were also drastically increased in TRIC37°C group after REBOA. TRIC12°C significantly downregulated the pro-inflammatory cytokines. In contrast, TRIC12°C significantly upregulated the levels of anti-inflammatory cytokines IL-4 and IL-10 after REBOA. Amazingly, the mortality rate was 100% in TRIC37°C group whereas 0% in TRIC12°C group after REBOA. Directly cooling the intestine offered exceptional protection of the abdominal organs from IRI and SIRS, switched from a harmful pro-inflammatory to a reparative anti-inflammatory response, and mitigated mortality in the rat model of REBOA management of lethal hemorrhage.

## Introduction

Noncompressible torso hemorrhage carries a high mortality rate^1^. Resuscitative endovascular balloon occlusion of the aorta, or REBOA, is a technique to manage torso hemorrhage^2–4^. This technique utilizes an occlusive balloon catheter inserted via the femoral artery into the descending aorta proximal to the injury site. Inflation of the balloon prevents the lethal hemorrhage by obstructing the aortic forward flow, but at the expense of distal abdominal organ ischemia^5^. Consequentially, sustained use of REBOA greater than 20–30 min leads to significant abdominal organ ischemia–reperfusion injury (IRI) and mortality^6^. Among abdominal organs, the intestines are the most damaged organ following the REBOA use^6^. For that reason, its clinical application remains limited^7^. Therefore, developing monitoring tools for abdominal organ IRI and therapies to prolong intestinal tissue tolerance to ischemia may be crucial to improving the clinical utility of REBOA.

Living tissue is highly susceptible to IRI under normothermic conditions. Ischemia leads to severe brain injury in under 5–10 min and can lead to significant dysfunction of the heart, intestines, liver, and kidneys in 10–20 min^8–11^. Severe intestinal IRI is often followed by a systemic inflammatory response, known as systemic inflammatory response syndrome (SIRS). Hypothermia is the most effective treatment currently known to protect the vital organs from IRI and SIRS^10,12,13^.

The objectives of this study were to investigate whether blood-based biomarkers can be used to indicate the IRI and SIRS and whether direct intestinal cooling can mitigate this damage in the rat model of REBOA management of lethal hemorrhage.

## Materials and methods

This study was carried out in compliance with the ARRIVE guidelines. All methods were carried out in accordance with relevant guidelines and regulations. The detailed descriptions of the materials and animal models are provided in Supplement Materials and Methods.

### Ethics statement

The animal protocol was approved by the Animal Use and Care Committee at the University of Maryland School of Medicine and conforms to the Guide for the Care and Use of Laboratory Animals by the United States National Institutes of Health (NIH Publication No. 85-23, 1996).

### Animal model

**Figure 1:**
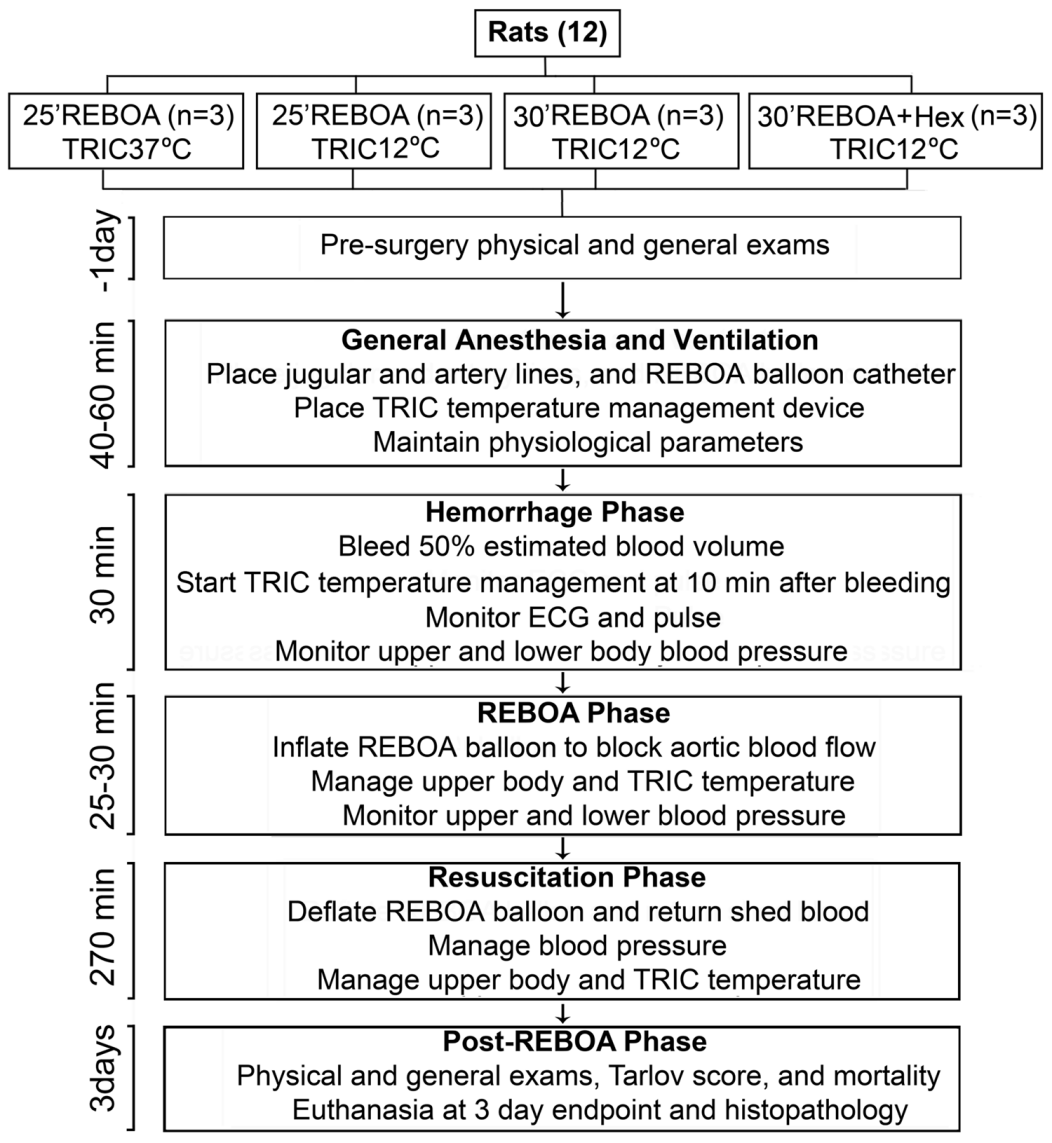
The flow chart and timeline of the study. The timeline was indicated on the left. Rats were subjected to 30 min hemorrhage followed by inflation of the REBOA balloon to occlude the descending aorta for either 25 min or 30 min. At the end of the REBOA phase, the withdrawn blood was returned, and the balloon was deflated to start the post-REBOA phase. TRIC temperature was kept either at 37 °C (TRIC37°C) or 12 °C (TRIC12°C) starting from 10 min after the beginning of the bleeding for a period of 270 min post-REBOA. In the 30 min REBOA + TRIC12°C + 0 °C Hex group, to further boost the abdominal tissue cooling, 5 ml of ice-cold (0 °C) Hextend solution (6% Hetastarch in Lactated Ringers) was quickly administered (within 1 min) via the tail arterial catheter immediately after inflation of the REBOA balloon. *REBOA* resuscitative endovascular balloon occlusion of the aorta, *TRIC* TransRectal Intra-Colon (temperature management device); *Hex* Hextend (6% Hetastarch in Lactated Electrolyte solution); and *ECG* electrocardiogram.

Figure 1 shows the flowchart and timeline of key events of this study. Male Sprague Dawley rats about 450 g were obtained from Charles River (Boston, USA). They were randomly pre-assigned identification numbers before the procedures. They were intubated, ventilated, and anesthetized throughout the procedure period. All survival surgery was performed by using aseptic techniques. Small incisions were made for inserting: (i) the inferior vena cava catheter line via the jugular vein in the neck for withdrawing blood; (ii) the right axillary arterial catheter for recording the proximal mean arterial pressure (MAP); (iii) the tail arterial catheter for recording the distal MAP; and (iv) a 2F Fogarty balloon catheter (Edwards Life Sciences, Irvine, USA) via the right femoral artery into the descending thoracic aorta for the implementation of REBOA. A TransRectal Intra-Colon (TRIC) temperature management device (see Supplement Materials and Methods) was inserted via the rectum into the descending colon into approximately 10 cm from the anus. A temperature probe was inserted into the descending colon to indicate the “intra-colon temperature.” Another temperature probe was inserted into the esophagus to indicate the upper body temperature. A final temperature probe was inserted into the bladder via the urinary tract to measure the bladder temperature to represent the abdominal cavity temperature.

The rat hemorrhage model was produced by withdrawing about 50% of the estimated total blood volume (EBV) via the inferior vena cava catheter (Supplement Materials and Methods). Animals were bled over a 30 min period in an exponential manner based on the method described by Park et al.^14^. The 30-min blood withdrawing period was based on our pilot study which showed that withdrawing 50% of the EBV from the jugular—inferior vena cava catheter could take about 20–30 min.

The complete REBOA was confirmed by the difference between proximal and distal pressure. The 50% of EBV hemorrhage led to both the proximal and distal blood pressure to a 25–30 mmHg range (Supplemental Tables 3 and 4). Inflation of the REBOA balloon led to a sharp increase in the proximal MAP from 25 to > 90 mmHg while a sharp drop of the distal MAP from 25 to about 10–20 mmHg in all groups. The results are consistent with the pressure changes in other complete REBOA animal models such as the swine model^14^.

The TRIC device was activated at 10 min after blood withdrawal. Ice-cold water was circulated through the TRIC device to reduce the colonic wall temperature to 12ºC in the TRIC12ºC groups. Water warmed to 36–37ºC was circulated in a same manner to maintain a colonic wall temperature of 37ºC in the TRIC37ºC group. The upper body temperature was maintained via a warmed pad and an overhead lamp as close to 37 °C as possible in all groups. Following the 30-min hemorrhage period, the REBOA balloon was inflated to occlude the descending aorta for a period of either 25 or 30 min. At the end of the REBOA phase, the withdrawn blood was returned, and the balloon was deflated to start the post-REBOA reperfusion phase. During the post-REBOA phase, the TRIC device remained active for a total of 270 min.

In the TRIC12°C group, rewarming was initiated at 180 min after REBOA, which was done by gradually increasing the temperature of the circulating water in the TRIC catheter to achieve a rewarming rate 0.15 °C/min as measured by the esophageal temperature until a target range of 30 °C was reached. The TRIC device was then removed, anesthesia discontinued, and the rats were returned to their cages. In the TRIC37°C group, the TRIC device was activated in the same manner and for the same period as those in TRIC12°C group but circulated with 37 °C water during the entire period.

The intra-colon and esophageal temperature (Supplemental Table 1 and 2), as well as the proximal and distal MAPs (Supplemental Table 3 and 4) were continuously recorded throughout the experimental period by Powerlab 16-channel data acquisition system (ADInstruments, Mountain View, CA, USA). The arterial blood gas (ABG) (pH, pCO2, pO2, electrolytes, lactate, base deficit, bicarbonate, glucose, and potassium) were measured via an ABL90 FLEX blood gas analyzer (Radiometer, USA). The prothrombin time (PT) and international normalized ratio (INR) was measured via the Coag-Sense PT/INR Professional System according to manufacturer instructions (CoaguSense, Inc. Fremont, CA, USA).

Blood samples were collected before blood withdrawal (pre-hemorrhage phase) at 0 min and then at 90 and 180 min after the end of REBOA deployment (referred to as the post-REBOA phase or post-REBOA hereafter). All animals were included for analysis except for one rat that died due to an unanticipated anesthesia event and was later replaced with another rat. The animal inclusion and exclusion criteria were predefined and not determined on a post-hoc basis.

### Experimental groups

The key events of the study are shown in Fig. 1. The following experimental groups were used: (i) 25 min REBOA + TRIC37°C (n = 3); (ii) 25 min REBOA + TRIC12°C (n = 3); (iii) 30 min REBOA + TRIC12°C (n = 3); and (iv) 30 min REBOA + TRIC12°C + 0 °C Hex (n = 3). In the 30 min REBOA + TRIC12°C + 0 °C Hex group, to further boost the abdominal tissue cooling, 5 ml of ice-cold (0 °C) Hextend solution (6% Hetastarch in Lactated Ringers) was quickly administered (within 1 min) via the tail arterial catheter immediately after inflation of the REBOA balloon. The rationale for using TRIC37°C as the normothermic control was to keep the two groups in the same intra-colon management condition (except temperature) and to prevent any possible effect created by the placement of the TRIC catheter inside the colon (such as pressure due to the catheter expansion). Our pilot study showed that the TRIC12°C offered virtually 100% survival rate while TRIC37°C resulted in 100% mortality in the rat model after 20–25 min REBOA management of lethal hemorrhage. The power analysis was performed to determine the number of animals required for each experimental group. Based on the mortality rate of 100% in the 25 min REBOA + TRIC37°C and 10% in the 25 min REBOA + TRIC12°C group, three rats are required for each experimental group for detecting a statistical difference with a power of 0.80, an alpha of 0.05 and a beta of 0.2.

### Measurement of plasma I-FABP/FABP2 and endotoxin

Intestinal injury was assessed by the plasma levels of fatty-acid binding protein (I-FABP), also known as FABP2 (Novus Biologicals, CO, USA), and endotoxin^15^ (Thermo Fisher Scientific, Rockford, IL, USA) according to the manufacturer’s instructions.

### Immunoassay for organ damage biomarkers

Liver injury was assessed by measuring the plasma levels of aspartate aminotransferase (AST) and fatty acid-binding protein 1 (FABP1). Cardiac injury was evaluated by the elevation of the level of plasma/serum cardiac troponin I (cTnI). Kidney injury was assayed by the elevation of the level of plasma/serum creatinine. The plasma levels of FABP1 (Novus Biologicals, CO, USA), creatinine (Sigma, St. Louis, MO, USA), and troponin I (Cambridge, MA, USA) were determined by the assay kits according to the manufacturer’s instructions.

### Cytokine assay

The plasma levels of pro-inflammatory cytokines (TNFα, IL-1β, and IL-17F) and anti-inflammatory cytokines (IL-4 and IL-10) were determined by using ELISA kits according to manufacturers’ instructions (R&D Systems, MN, USA).

### Statistical analysis

Data are expressed as mean ± standard error of the mean (SEM). One-way ANOVA followed by Tukey post-hoc test for statistical analysis of values of tissue injury biomarkers and cytokines. The log-rank test for survival rate analysis. Chi-Square test for mortality rate. GraphPad Prism version 7.00 for Windows was used (GraphPad Software, La Jolla, California, USA). *p* < 0.05 was considered a statistically significant difference.

## Results

### TRIC12°C reduces REBOA-induced intestinal injury

**Figure 2:**
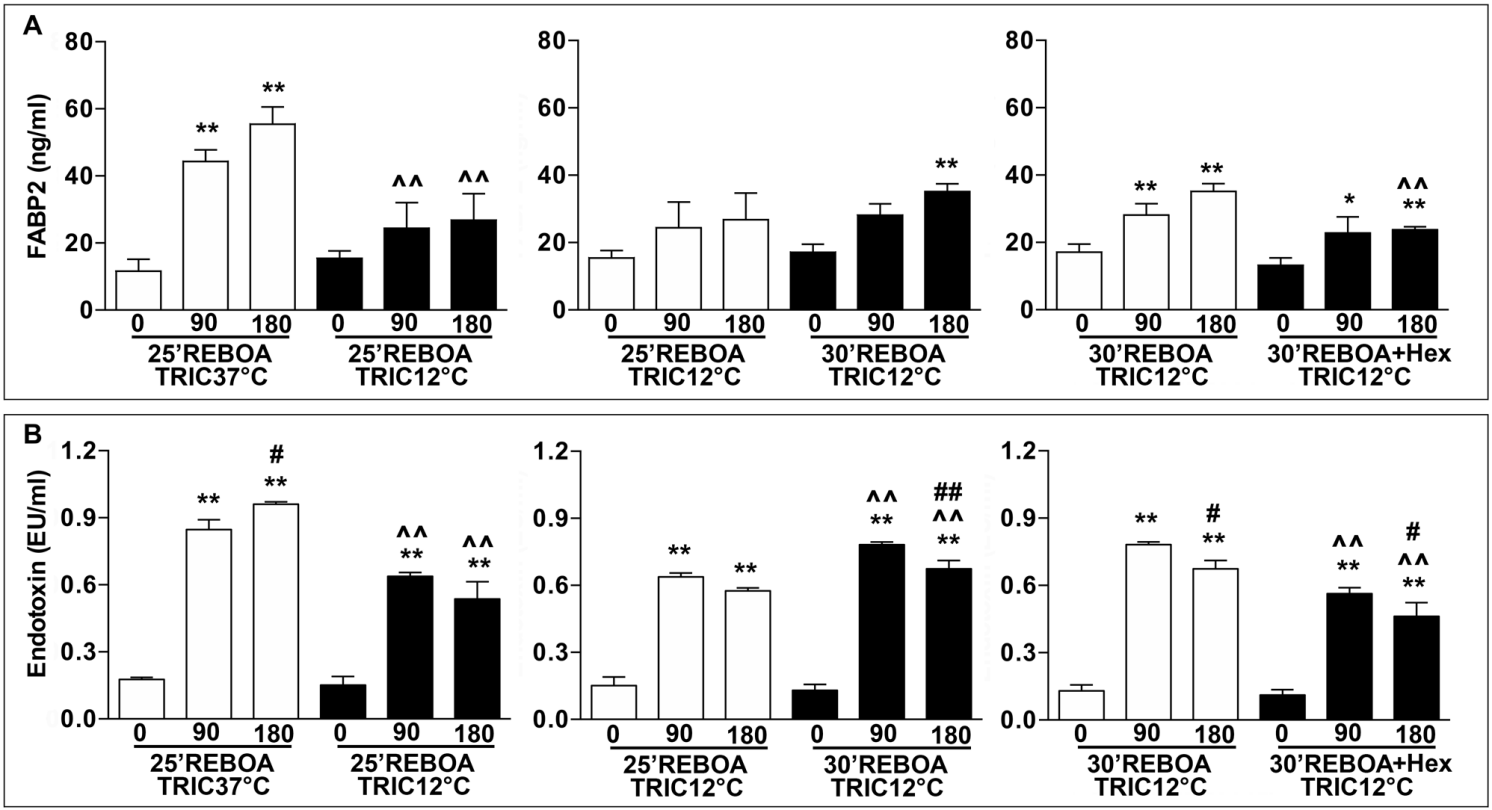
(**A**) Plasma level of intestinal fatty acid binding protein (I-FABP or FABPI). (**B**) Plasma level of endotoxin. The experimental groups were (i) 25 min REBOA + TRIC37°C (n = 3); (ii) 25 min REBOA + TRIC12°C (n = 3); (iii) 30 min REBOA + TRIC12°C (n = 3); and (iv) 30 min REBOA + TRIC12°C + 0 °C Hex (n = 3). Rats were subjected to 30 min hemorrhage followed by inflation of the REBOA balloon for either 25 min (25 min REBOA) or 30 min (30 min REBOA). TRIC temperature was kept either at 37 °C (TRIC37°C) or 12 °C (TRIC12°C). At the end of the REBOA, the withdrawn blood was returned, and the balloon was deflated to start the post-REBOA reperfusion phase. In the 30 min REBOA + TRIC12°C + 0 °C Hex group, 5 ml of ice-cold (0 °C) Hextend solution was quickly administered via the tail arterial catheter immediately after inflation of the REBOA balloon. Blood plasma samples were collected at pre-hemorrhage (0), as well as at 90 and 180 min after deflation of the REBOA balloon (post-REBOA). Data are expressed as mean ± SEM, one-way analysis of variance (ANOVA) followed by Tukey test to determine statistical significance. *p < 0.05 and **p < 0.01, 0 min vs. 90 or 180 min; ^*p* < 0.05 and ^^*p* < 0.01, 90 or 180 min vs. 90 or 180 min; and #*p* < 0.05 and ##*p* < 0.01, 90 vs. 180 min.

Figure 2A shows that, relative to the pre-hemorrhage level (0), the plasma FABP2 levels were significantly increased by about 4.5-fold at 90 min and more than fivefold at 180 min post-REBOA in TRIC37°C rats. The focal intestinal cooling (25 min REBOA + TRIC12°C) significantly reduced the plasma FABP2 levels at both 90 (about 44.92%) and 180 (about 51.55%) min post-REBOA, compared to the same time points of the TRIC37°C group (25 min REBOA + TRIC37°C) (Fig. 2A). An increase in REBOA duration by 5 min (30 min REBOA + TRIC12°C) insignificantly increased the plasma FABP2 level, compared to that of the 25 min REBOA + TRIC12°C group (Fig. 2A). On the contrary, the infusion of 0 °C HEXTED (30 min REBOA + TRIC12°C + 0 °C Hex) significantly decreased the blood FABP2 level by about 32.36%, relative to that of the 30 min REBOA + TRIC12°C group.

### TRIC12°C Reduces REBOA-induced Sepsis

Figure 2B shows that, relative to the pre-hemorrhage level (0), the plasma endotoxin levels were significantly increased by about fourfold at 90 min and more than 4.5-fold at 180 min post-REBOA in TRIC37°C rats. The focal intestinal cooling (25 min REBOA + TRIC12°C) significantly reduced the plasma endotoxin levels at both 90 min and 180 min post-REBOA by about 50%, compared to the same time points of the TRIC37°C group (25 min REBOA + TRIC37°C) (Fig. 2B). An increase in REBOA duration by 5 min (30 min REBOA + TRIC12°C) significantly increased in the plasma endotoxin level by about 22.49% (*p* < 0.01), compared to that of the 25 min REBOA + TRIC12°C group (Fig. 2B). The infusion of ice-cold HEXTED (30 min REBOA + TRIC12°C + 0 °C Hex) further decreased the blood endotoxin level by about 31.45% (*p* < 0.01), relative to that of the 30 min REBOA + TRIC12°C group.

### TRIC12°C reduces REBOA-induced organ injury

**Figure 3:**
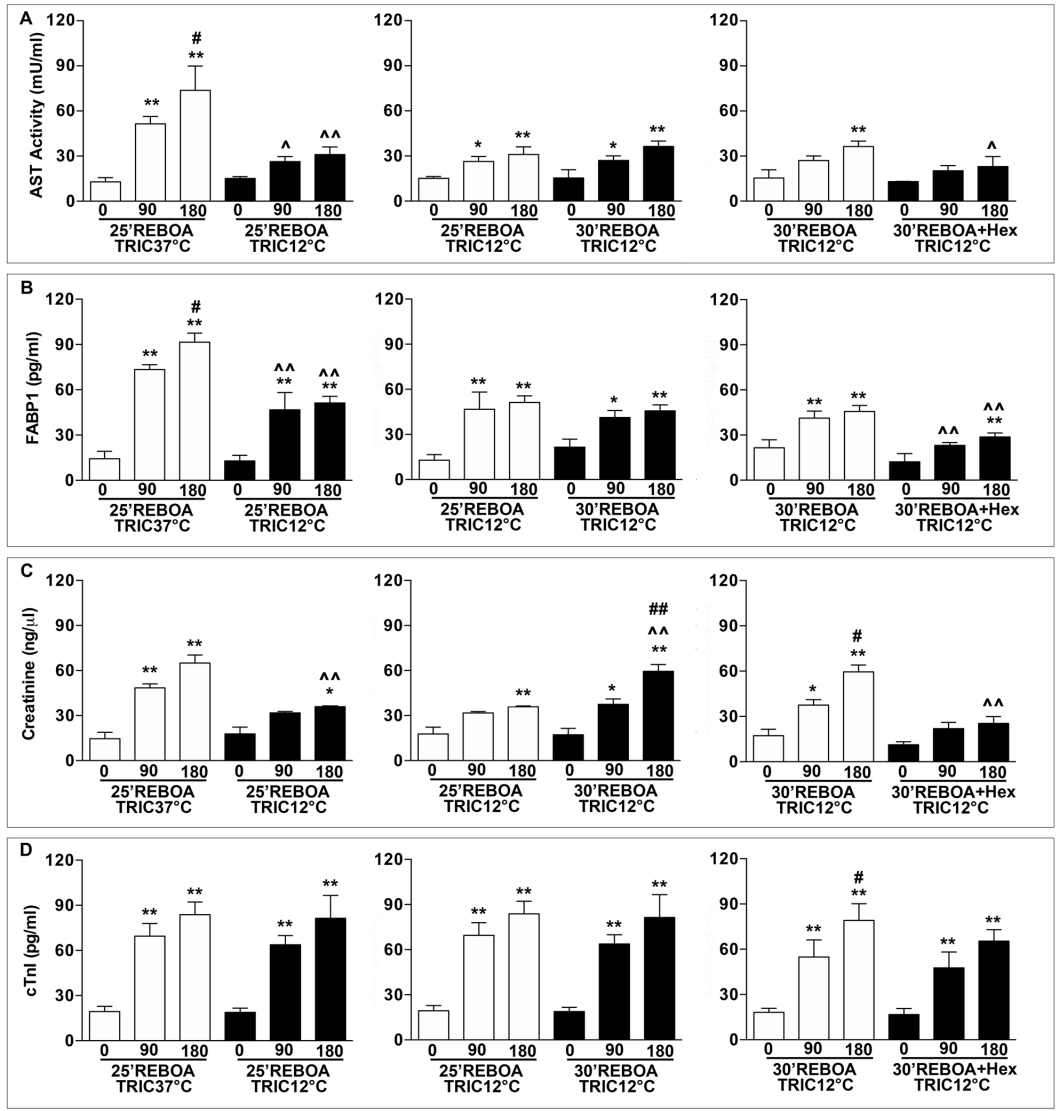
(**A**) Plasma level of aspartate amino-transferase (AST). (B) Plasma level of fatty acid-binding protein 1 (FABP-1). (**C**) Plasma level of Creatinine. (D) Plasma level of Troponin I (cTnl). The same animal groups and blood sample collection schedule as in Fig. 2 were used. Data are expressed as mean ± SEM, one-way ANOVA followed by the Tukey test was used to determine statistical significance. *p < 0.05 and **p < 0.01, 0 min vs. 90 or 180 min; ^*p* < 0.05 and ^^*p* < 0.01, 90 or 180 min vs. 90 or 180 min; and #*p* < 0.05 and ##*p* < 0.01, 90 vs. 180 min.

Figure 3 shows the plasma concentrations of the organ injury biomarkers. Figure 3A,B reveal that, relative to the pre-hemorrhage level (0 min), the plasma AST and FABP1 levels were significantly increased at 90 (fourfold) and 180 (sixfold) min post-REBOA in 25 min REBOA + TRIC37°C rats. In comparison, the AST and FABP1 levels in 25 min REBOA + TRIC12°C group were significantly reduced by > 50% at both 90 min and 180 min post REBOA relative to the same time points of the 25 min REBOA + TRIC37°C group. When the REBOA duration was increased from 25 to 30 min (30 min REBOA + TRIC12°C group), there was no significant additional increase in either the AST or FABP1. However, the infusion of ice-cold HEXTED (30 min REBOA + TRIC12°C + 0 °C Hex) further decreased the blood AST or FABP1 levels by about 36.8% (*p* < 0.01), relative to that of the 30 min REBOA + TRIC12°C group.

Figure 3C shows the levels of creatinine. There was a 2.5-fold increase in the creatinine in the 25 min REBOA + TRIC37°C group at 90 min and a threefold increase at 180 min in the post-REBOA phase, relative to the creatinine level at the pre-hemorrhage level (0 min). When TRIC12°C was applied, there was non-significant decrease in the creatinine level by about 34.41% (*p* > 0.05) at 90-min and a remarkable decrease by about 44.8% (*p* < 0.01) at 180-min post-REBOA time point. The level of creatinine was significantly higher at 180 min post-REBOA in 30 min REBOA + TRIC12°C group than that in 25 min REBOA + TRIC12°C group. The level of creatinine was significantly lower at 180 min post-REBOA in 30 min REBOA + TRIC12°C + 0 °C Hex group than that in 30 min REBOA + TRIC12°C group.

Figure 3D illustrates the concentration of serum cTnI. Relative to the pre-hemorrhage level (0 min) the levels of cTnI were significantly increased at both 90 and 180 min post-REBOA in all four groups. There were no statistical differences in the levels of cTnI among the four experimental groups, although the levels of cTnI in TRIC12°C groups tended to be lower than in TRIC37°C group.

### TRIC12°C reduces pro-inflammatory cytokines

**Figure 4:**
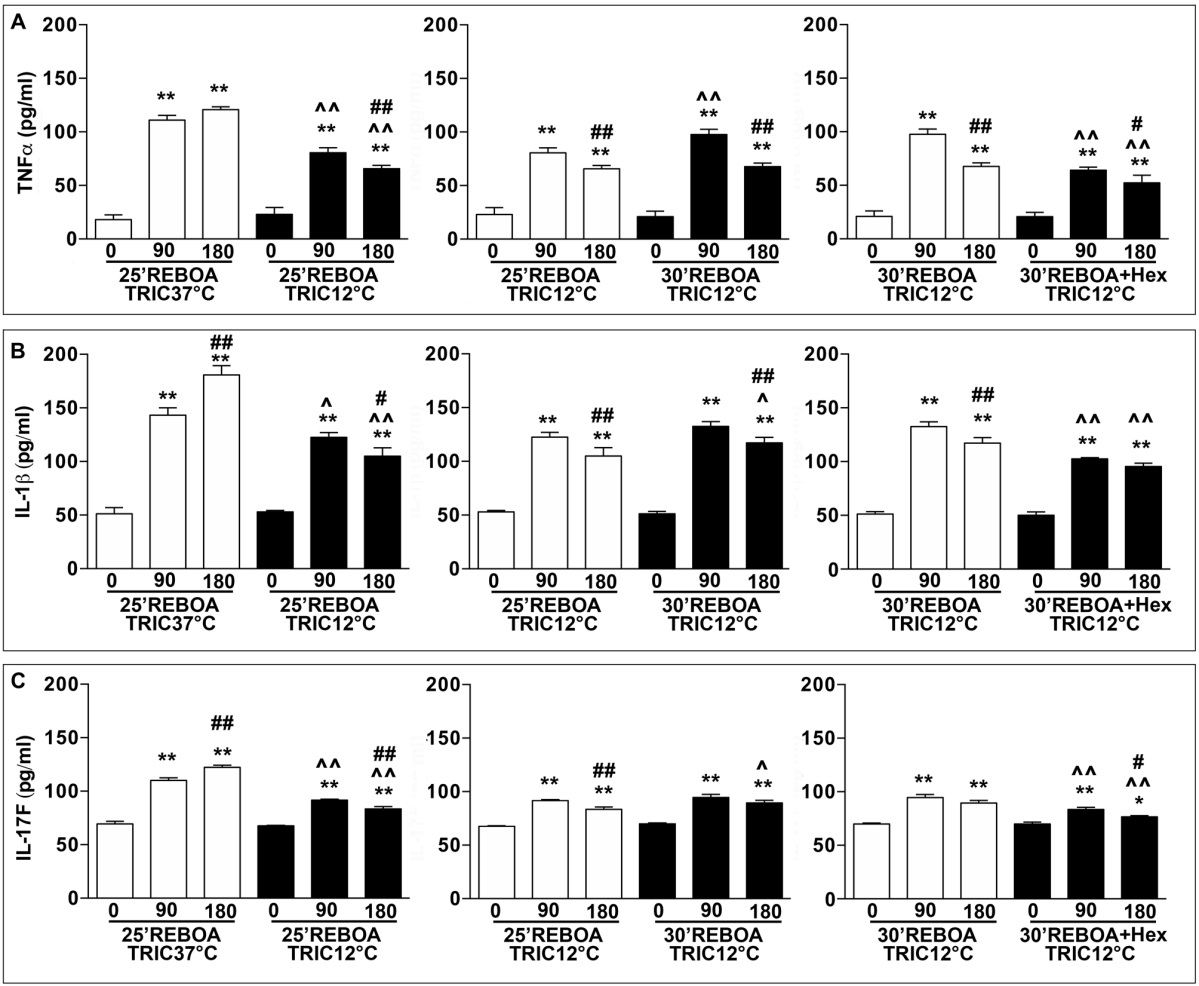
(**A**) Plasma level of TNFα. (**B**) Plasma level of IL-1β. (**C**) Plasma level of IL-17F. The same animal groups and blood sample collection schedule as in Fig. 2 were used. Data are expressed as mean ± SEM, one-way ANOVA followed by the Tukey test was used to determine statistical significance. *p < 0.05 and **p < 0.01, 0 min vs. 90 or 180 min; ^*p* < 0.05 and ^^*p* < 0.01, 90 or 180 min vs. 90 or 180 min; and #*p* < 0.05 and ##*p* < 0.01, 90 vs. 180 min.

Figure 4 displays the plasma levels of pro-inflammatory cytokines (TNFα, IL-1β, and IL-17F). Relative to those of the pre-hemorrhage (0 min), the plasma levels of TNFα, IL-1β, and IL-17F were significantly increased at 90 (5.5-fold for TNFα, 2.8-fold for IL-1β, and 1.5-fold for IL-17F) and 180 min (sixfold for TNFα, fourfold for IL-1β, and twofold for IL-17F) post-REBOA in the 25 min REBOA + TRIC37°C groups. The plasma levels of TNFα, IL-1β, and IL-17F in the 25 min REBOA + TRIC12°C rats were significantly reduced by an average of 27.52% for TNFα, 14.53% for IL-1β, and 16.67% for IL-17F, compared to 25 min REBOA + TRIC37°C group (Fig. 4A–C). An increase in 5 min in the REBOA duration (30 min REBOA + TRIC12°C) showed a significant increase in the plasma level of TNFα but had minimal effect on the plasma levels of IL-1β, and IL-17F, compared to those of the TRIC12°C + 25 min REBOA group (Fig. 4A–C). The infusion of ice-cold HEXTED (30 min REBOA + TRIC12°C + 0 °C Hex) further decreased the plasma levels of TNFα (about 34.38%), IL-1β (about 22.64%), and IL-17F (about 11.86%), respectively, relative to those of the TRIC12°C + 30 min REBOA group (Fig. 4A–C).

### TRIC12°C increases anti-inflammatory cytokines

**Figure 5:**
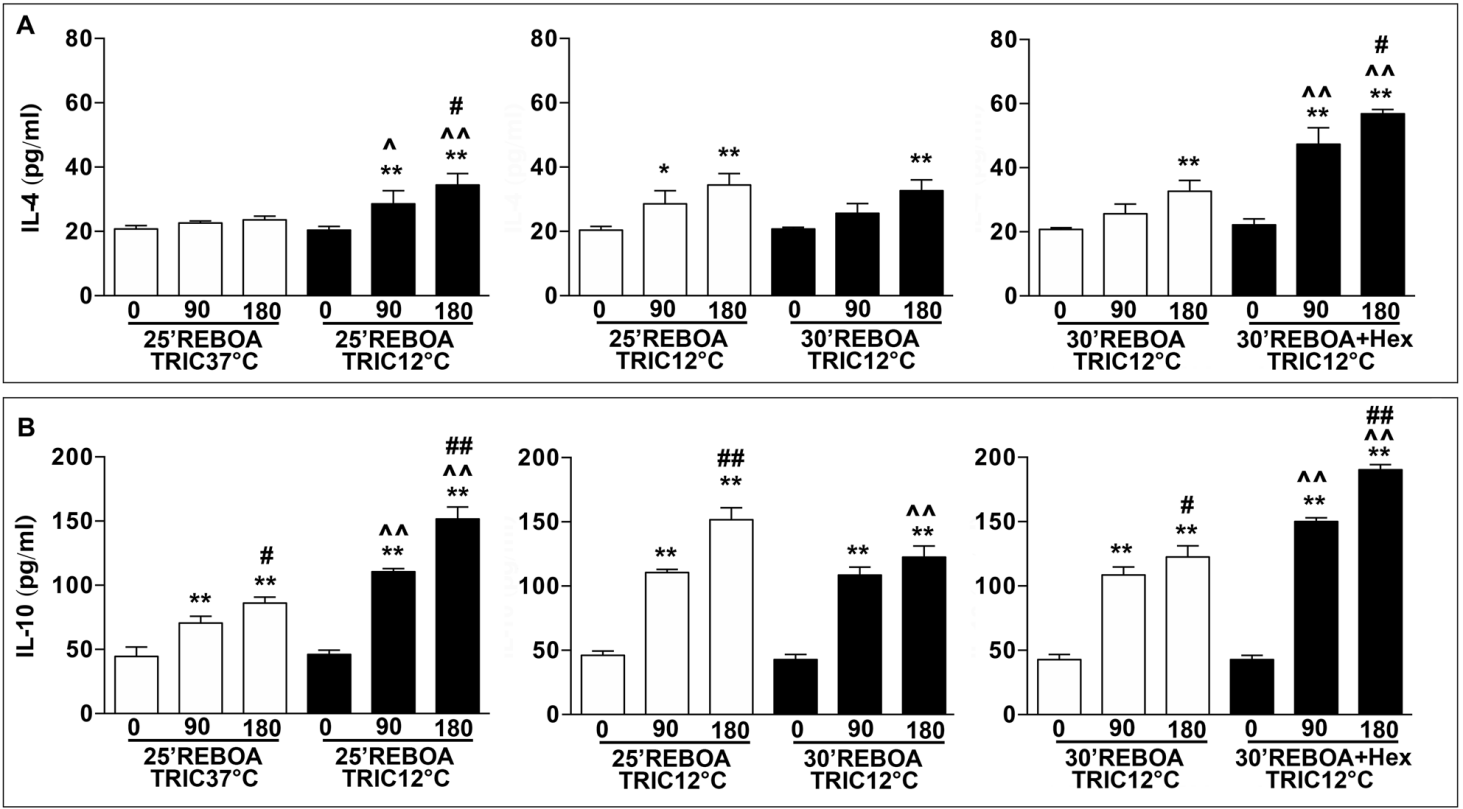
(**A**) Plasma level of IL-4. (**B**) Plasma level of IL-10. The same animal groups and blood sample collection schedule as in Fig. 2 were used. Data are expressed as mean ± SEM, one-way ANOVA followed by the Tukey test was used to determine statistical significance. *p < 0.05 and **p < 0.01, 0 min vs. 90 or 180 min; ^*p* < 0.05 and ^^*p* < 0.01, 90 or 180 min vs. 90 or 180 min; and #*p* < 0.05 and ##*p* < 0.01, 90 vs. 180 min.

Figure 5 shows the plasma levels of anti-inflammatory cytokines (IL-4 and IL-10). Relative to levels of the pre-hemorrhage (0 min), the plasma levels of IL-4 were not significantly increased, while IL-10 was significantly increased at 90 and 180 min (by about 1.5 and twofold) post-REBOA in the 25 min REBOA + TRIC37°C group. In comparison, the plasma levels of IL-4 and IL-10 in 25 min REBOA + TRIC12°C group were significantly upregulated by about 45% for IL-4, and about 76% for IL-10, compared to 25 min REBOA + TRIC37°C group (Fig. 5A,B). An increase in REBOA duration by 5 min (30 min REBOA + TRIC12°C) significantly decreased in the plasma level of IL-4 and IL-10 at 180 min post-REBOA, compared to those of the TRIC12°C + 25 min REBOA group (Fig. 5A,B). The infusion of ice-cold HEXTED (30 min REBOA + TRIC12°C + 0 °C Hex) significantly upregulated the plasma levels of IL-4 (about 76%) and IL-10 (about 55%), respectively, relative to those of the TRIC12°C + 30 min REBOA group (Fig. 5A,B).

### TRIC12°C reduces mortality

**Figure 6:**
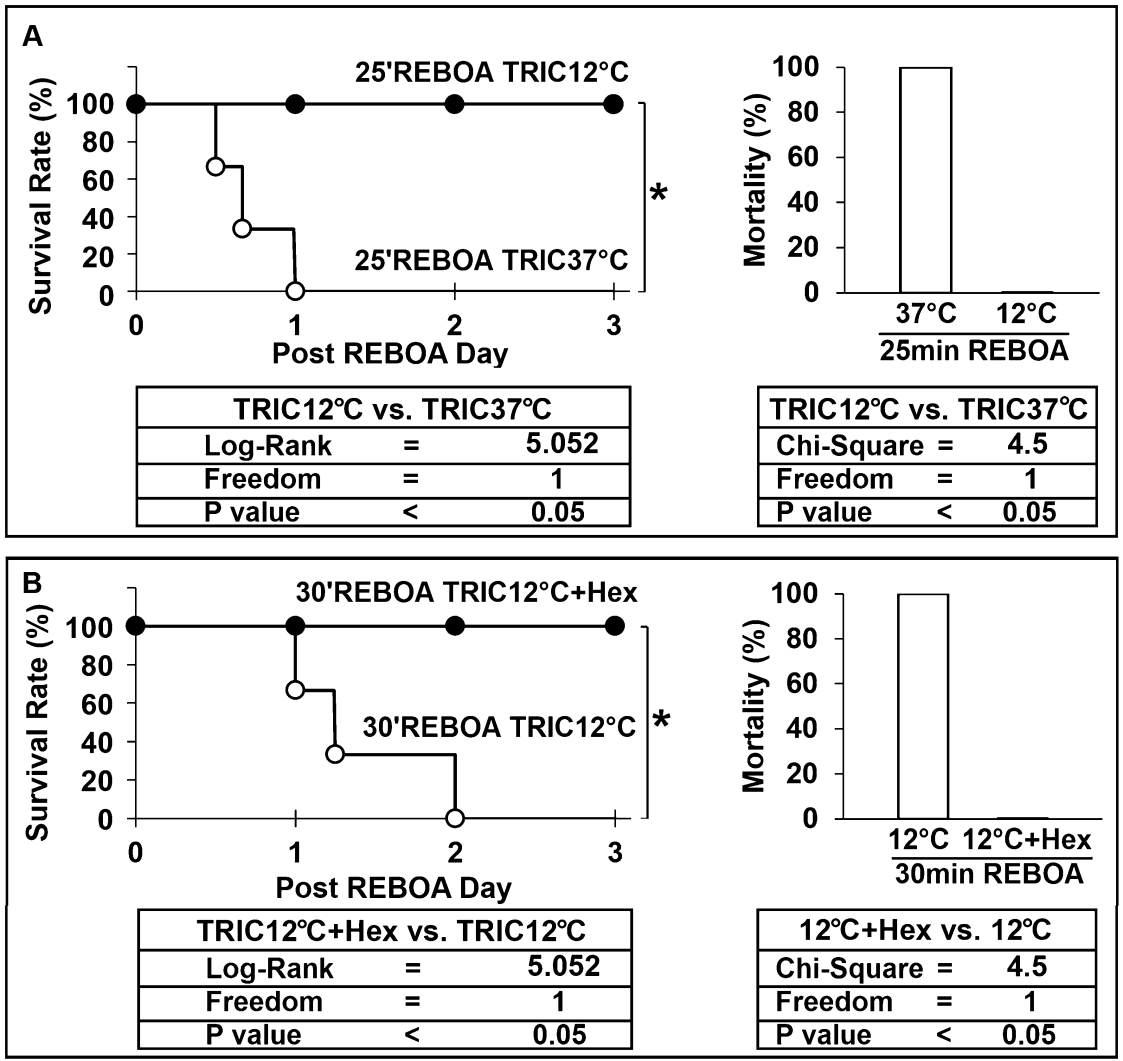
Survival rate and Mortality. (**A**) 25 min REBOA + TRIC37°C (n = 3) vs. 25 min REBOA + TRIC12°C (n = 3). (**B**) 30 min REBOA + TRIC12°C (n = 3 vs. 30 min REBOA + TRIC12°C + 0 °C Hex (n = 3). Statistical analyses of the data are shown in the tables below. Mortality data are expressed as mean ± SEM. Log Rank Test was used for survival rate statistical analysis. Pearson-Chi Square test was used for mortality rate statistical analysis.

The survival rate was compared among the experimental groups used in this study (Fig. 6). All rats died within 24 h in the 25 min of REBOA + TRIC37°C group (Fig. 6A). In contrast, all rats survived until the endpoint in the 25 min of REBOA + TRIC12°C group (Fig. 6A). All rats died within 2 days in the 30 min of REBOA + TRIC12°C group, while all rats survived to the endpoint in the 30 min of REBOA + TRIC12°C + 0 °C Hex group (Fig. 6B). Therefore, the mortality rate was 100% for 25 min REBOA + TRIC37°C rats, 0% for 25 min REBOA + TRIC12°C rats, 100% for 30 min REBOA + TRIC12°C rats, and 0% for 30 min REBOA + TRIC12°C + 0 °C Hex rats.

### Effect of TRIC12°C on PT/INR

**Figure 7:**
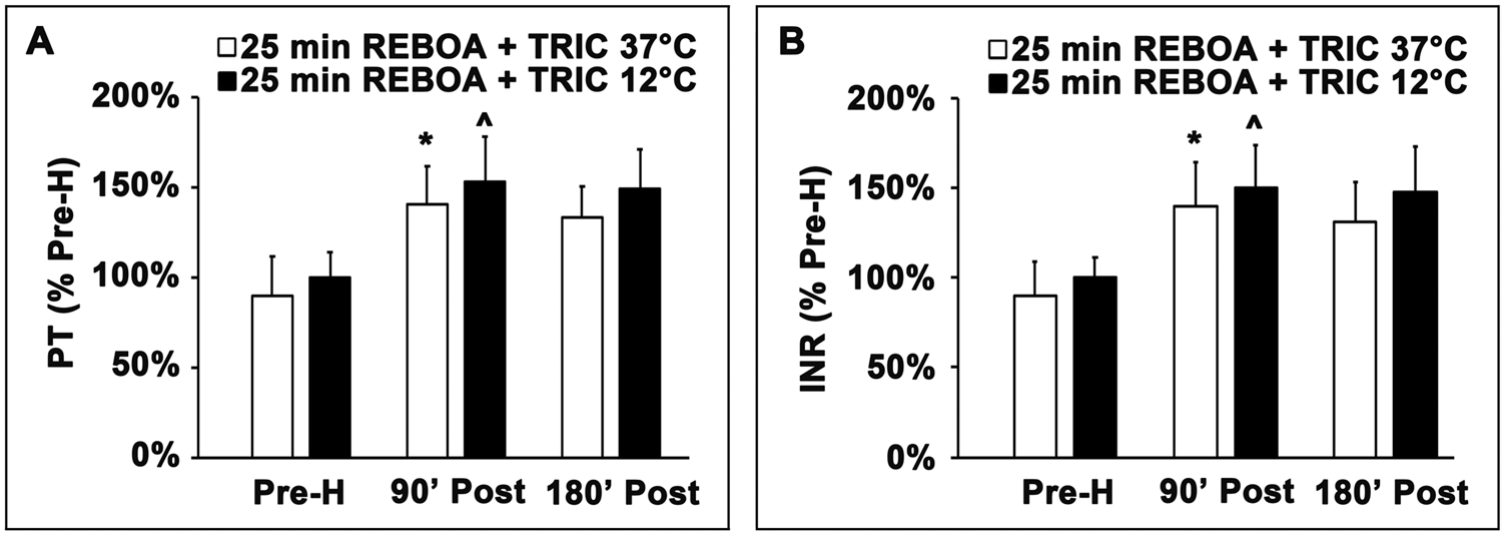
Effects of TRIC12°C cooling on PT/INR: (**A**) Prothrombin time (PT). (**B**) International normalized ratio (INR). PT and INR were measured or calculated at pre-hemorrhage (0), as well as at 90 and 180 min after deflation of the REBOA balloon (post-REBOA) in the TRIC12°C and TRIC37°C groups. Data are expressed as mean ± SEM (n = 3 for TRIC12°C; n = 3 for TRIC37°C). *p < 0.05, 0 vs. 90 min of the TRIC12°C; ^*p* < 0.05, 0 vs. 90 min of the TRIC37°C group. The PT and INR values tended to be lower in the TRIC37°C group than in the TRIC12°C group, but the differences were statistically insignificant. two-tailed *t* test.

Figure 7 shows the PT/INR values among the experimental groups. Relative to the pre-hemorrhage (Pre-H) value, the PT/INR value was significantly increased at 90 min (90’ Post) post-REBOA and then decline slightly at 180 min (180’ Post) among all cooling and non-cooling groups, and thus probably as a result of the use of heparin during the surgical procedures (Fig. 7A,B). However, the PT/INR value tended to be persistently higher in TRIC12°C groups than in the TRIC37°C group, although the changes were insignificant (Fig. 7A,B). The insignificant changes in PT/INR values between cooling and non-cooling groups were likely because that the study was not powered to detect the significant difference.

## Discussion

This study demonstrates that after 25 min REBOA management of 50% bleeding, all normothermic rats died (25 min REBOA + TRIC37°C group). Strikingly, none of the rats undergoing cooling by TRIC (25 min REBOA + TRIC12°C group) died. This difference was reflected in the plasma levels of organ injury biomarkers and pro-inflammatory cytokines, which were all drastically elevated in the normothermic group (25 min REBOA + TRIC37°C). In comparison, direct TRIC cooling the intestines at 12 °C significantly reduces the plasma levels of the organ injury biomarkers and the injurious pro-inflammatory cytokines. TRIC12°C also upregulated the plasma levels of anti-inflammatory cytokines. Furthermore, TRIC12°C combined with a quick arterial infusion of ice-cold HEXTEND solution (TRIC12°C + 0 °C HEX) offered an additional and staggering reduction of the key organ injury biomarkers and injurious pro-inflammatory cytokines. These results demonstrate that both TRIC12°C and TRIC12°C + 0 °C HEX provide a robust protection of abdominal organs from IRI and SIRS.

### Post-REBOA mortality

This study showed that directly cooling the intestine offered exceptional protection against mortality in the rat model of REBOA management of lethal hemorrhage. The results are consistent with a recent study from our laboratory, which showed that TRIC12°C reduced mortality after 20 min of REBOA management of (50% EBV) lethal hemorrhage^16^. The power analysis of the mortality of the previous study between cooling and non-cooling groups showed that 3 rats per experimental group were required in this study.

### Post-REBOA organ injury biomarkers

The changes in the plasma levels of the abdominal organ injury biomarkers are consistent with the several histopathological injuries to the intestines, liver, and kidneys during the post-REBOA phase in both the rat and swine model^16^. Identifying readily accessible or point-of-care blood-based biomarkers that can predict clinical trajectory during early the post-REBOA phase is clinically relevant. These point-of-care REBOA “companion” biomarkers may allow for the personalization of the REBOA management duration so that vascular and trauma surgeons can individualize REBOA occlusion time to complete their interventions. The results of this study suggest that endotoxin and FABP2/I-FABP may be used for indicating intestinal damage, creatinine for kidney damage, and AST and FABP1/L-FABP for liver damage during the post-REBOA phase. However, among these biomarkers, only endotoxin and creatinine might predict the mortality. Plasma levels higher than 0.65 EU/ml for endotoxin and 40 ng/μl for creatinine correlated with 100% post-REBOA mortality, whereas all the survival animals had endotoxin significantly lower than 0.65 EU/ml (*p* < 0.05) and creatinine lower than 40 ng/μl (*p* < 0.05).

### Post-REBOA pro-inflammatory cytokine storm and SIRS

SIRS result from a surge in the release of pro-inflammatory cytokines. The term cytokine storm occurs when there is a persistent and uncontrolled release of these pro-inflammatory cytokines. This release of cytokines can occur due to severe tissue injury or response to a profound infection or foreign entity^17^. TNF-α, IL-1β, and IL-17F represent a typical array of pro-inflammatory cytokines. The plasma levels of the pro-inflammatory cytokines are significantly higher in TRIC37°C group than in the TRIC12°C groups after REBOA. However, within the TRIC12°C groups, the plasma levels of the pro-inflammatory cytokines were all relatively low, even though all the rats in the 30 min REBOA + TRIC12°C group died. This may suggest that, although the cooling inhibited the upregulation of pro-inflammatory cytokines, factors other than pro-inflammatory cytokines may also play a vital role in the survival. Our study provides evidence to suggest that both abdominal organ injuries and pro-inflammatory response contribute to post-REBOA mortality.

### TRIC activates the anti-inflammatory response after REBOA

This study further shows that directly cooling the intestine activated an anti-inflammatory response during the post-REBOA phase. This finding is consistent with reports that hypothermia can elicit a powerful anti-inflammatory reaction^18,19^. Previous results have shown that hypothermia upregulates the blood levels of IL-4 and IL-10 and can inhibit neutrophil aggregation and production of nitric oxide during endotoxemia^18^. Although IL-4 and IL-10 are key anti-inflammatory cytokines downregulating SIRS, we found that TRIC12°C increased in the plasma level of IL-10 more significantly than that of IL-4 (*p* < 0.05). A plasma IL-10 level higher than 108 pg/ml seemed to correlate with 100% survival (*p* < 0.05). Furthermore, the ratios of TNF-α/IL-10 < 0.8, IL-1β/IL-10 < 1.2, and IL-17F/IL-10 < 1.5 seemed to also correlate with 100% animal survival in our model.

### TRIC may be a better therapeutic hypothermia modality

The present study provides evidence that utilizing TRIC for cooling the intestine offers exceptional protection against fatal IRI and SIRS. There are currently two main therapeutic temperature management techniques: (i) external skin cooling and (ii) endovascular cooling. Both cooling techniques are highly inefficient, as cooling down an average-sized 65 kg human (65 kg × 65 mL blood/kg body weight = 4 L of human blood and its supplied tissue) from 37 to 30 °C would take several hours at a cooling rate about 2–4 °C/hour^12,20^. By contrast, the TRIC device can cool down the gut temperature from 37 to 10 °C in about 2–5 min because of its direct contact. This localized cooling also creates a temperature gradient in the surrounding organs while maintaining a tolerable upper body temperature range above 28 °C and avoids low temperature-induced fatal cardiorespiratory failure^21,22^. Therefore, TRIC cooling is not a systemic hypothermia, rather than a focal cooling technique to achieve the optimal cooling temperature (TRIC12°C) for the maximal protection of the intestines during the post-REBOA phase.

This study demonstrated that the level of endotoxin and FABP2/I-FABP were drastically increased during the post-REBOA phase, indicative of severe intestinal injury and the likely development of intraabdominal sepsis. In addition to sepsis, the intestines are equipped with the largest pool of macrophages and represent more than 70% of the entire immune system in the body^23,24^. Therefore, intestinal injury during the post-REBOA phase activates immune cells and macrophages, which propagates systemic inflammation resulting in SIRS. As a result, intestinal injury has been considered the “origin” or “motor” of the SIRS after lethal hemorrhage^25^. TRIC12°C significantly reduced the intestinal IRI as indicated by the lower plasma levels of endotoxins and FABP2/I-FABP. Therefore. cooling the intestines may equal to cooling the immune organ for the management of SIRS after REBOA management of lethal hemorrhage.

### Post-REBOA cold perfusion shows further protection

The cold perfusion may represent the most effective measure for preserving transplant organs outside the body^26^. However, it may not be clinically feasible to perform deep hypothermia in most living patients, as the vital organs, particularly the heart and lungs, require warm oxygenated blood to function properly^21,22^. Once the REBOA is deployed, it blocks the blood flow to the distal abdominal organs and provides an opportunity to perform the cold perfusion of the distal abdominal organs inside the body while having insignificant impact on the heart and lungs. In early organ transplantation studies, simple surface cooling and cold perfusion with heparinized blood were used to preserve transplant organs for transport^26^. It was later realized that the flushing out the blood cellular components with cold physiological electrolyte solutions followed by the static cold storage offered considerably better organ preservation^26^. Our study showed, in conjunction with TRIC focal cooling, the quick arterial infusion of ice-cold Hextend solution into the abdominal organs offered greater protection against post-REBOA fatal IRI and SIRS without adverse effects to the heart or lungs.

### Limitation of this study

Several limitations exist in this study. A limitation of the present study may be that the lethal hemorrhagic shock model was produced by bleeding 50% of the EBV without a primary traumatic injury. However, the implementation of REBOA is likely to stop the torso hemorrhage from the primary injury, thus reducing the risk of the enhanced ongoing bleeding caused by intra-colon cooling. Furthermore, some clinical situations may require the use of REBOA management of significant bleeding without a primary traumatic injury, such as aortic aneurysm injury and repair, or the unanticipated and uncontrolled bleeding during abdominal surgery. Therefore, the lethal hemorrhagic model (without severe tissue trauma) was often used in swine REBOA studies^14^.

There is an inverse relationship between temperature and prothrombin time (PT)^27^. As shown in Fig. 7, the PT/INR were moderately increased after REBOA, which was likely due to the use of heparin for the surgical preparation of the animal model. However, the increases in the PT/INR seemed somewhat more in TRIC12°C than in TRIC37°C group, which could be a result of TRIC cooling. The insignificant difference in PT/INR between TRIC12°C and TRIC37°C groups is likely because that the study was not powered to detect the difference. However, the PT/INR differences between the two groups appeared not as dramatic as what was expected at the intestinal 12 °C condition^27^. The smaller PT/INR differences between TRIC12°C and TRIC37°C groups are likely because, during implementation of REBOA, most of the body blood volume was circulating in the “warmer” upper body. As a result, TRIC intra-colon cooling may have a limited impact on the systemic coagulation cascade. Furthermore, implementation of REBOA might alleviate the cooling-induced bleeding risk in the abdominal organs. Nevertheless, coagulopathy after injury is complex. PT/INR may not sufficiently represent the derangements. Further investigation of broader coagulation parameters is needed to understand the effects of TRIC cooling on the potential coagulopathy after injury.

Another issue may be whether TRIC device can be used in humans as efficiently in rats. In a currently ongoing study of direct gut cooling in a swine model, we have demonstrated that TRIC directly cooling the gut can quickly reduce the intra-colon temperature from 37 to 12 °C within 10 min and bladder from 37 °C to about 25 °C within 30–40 min in a 45 kg naïve swine, while maintaining the esophageal upper body temperature in the tolerable range.

It also should be pointed out that TRIC cooling was initiated prior to the REBOA implementation in this study. Further animal study may be needed to investigate the scenario of the simultaneous implementation of both REBOA and TRIC cooling for mimicking patients who present in extremis and thus require prompt implementation of REBOA.

Additionally, the timescales of major biological events are different between rats and humans. For example, an average life expectancy is ~ 27 times shorter while the basal metabolic rate is ~ 6.4 times faster in rats than in humans^28^. However, the rat model is easily manageable, can be controlled in virtually identical experimental conditions and economical, while having the cardiovascular, organ injury biomarkers, and inflammatory response systems similar to those of humans. Therefore, the rat model may be suitable for the proof-of-concept studies. Significant efforts are needed for potential translating the proof-of-concept TRIC protection from the small animal model to the large animal model, and eventually to human applications.

### Potential mechanisms of anti-inflammatory effects of directly cooling intestines

A critical question may be why fast and deep cooling gut is more effective in reducing SIRS than cooling other organs in the body. In broad terms, the gut is comprised of three entities: the intestinal lumen commensal flora, the epithelium, and the mucosal immune system^29^. The release of intestinal luminal bacteria and toxins into the circulation due to gut IRI propagates systemic inflammation, and thus the gut is considered the most dangerous abdominal organ, and “origin” of SIRS under many severe medical conditions^29–34^. This may be consistent with the fact that SIRS can be induced by a long list of intestinal diseases and by all “leaky” intestinal injuries^30^. In order to prevent invasion of the intestinal microorganisms and toxins during transport of nutrients, the gut is equipped with the largest pool of immune cells representing more than 70% of the entire immune system and the largest population of macrophages in the body^29,30^. A recent study from our laboratory showed that the intestine was the most susceptible organ to IRI after implementation of REBOA^35^. The post-REBOA histopathological damage to the intestines was also observed in the present study (Supplemental Fig. 1). Consistently, the plasma I-FABP and endotoxin were drastically increased during the post-REBOA phase (Fig. 2). Evidence suggests that deep cooling the gut may be equivalent to cooling the major immune organ and thus is highly effective in lessening life-threaten SIRS after REBOA management of hemorrhage.

## Supplementary Information

Supplementary Information.
