# Supplementary material for: Focal intra-colon cooling reduces organ injury and systemic inflammation after REBOA management of lethal hemorrhage in rats
Source: Sci Rep. 2021 Jul 1;11:13696. doi: 10.1038/s41598-021-93064-4 (PMC8249469; doi:10.1038/s41598-021-93064-4)
Supplement: Supplementary file 1 — Supplementary Information. [file 41598_2021_93064_MOESM1_ESM.docx]

**Supplement Materials and Methods:**

**Title: Focal Intra-Colon Cooling Reduces Organ Injury and Systemic Inflammation After REBOA Management of Lethal Hemorrhage in Rats**

Authors: Awadhesh K Arya^1^ PhD, Kurt Hu^2^ MD, Lalita Subedi^1^ PhD, Tieluo Li^1^ MD, and Bingren Hu^1,3^* MD PhD

***Supplement Materials and Methods.***

***Materials:*** Aspartate aminotransferase (AST) activity (Cat# MAK055) and creatinine assay kit (Cat# MAK080), PBS, heparin and other chemicals were purchased from Sigma (Sigma, St. Louis, MO, USA). 2F Fogarty balloon catheters were purchased from Edwards Life Sciences (Irvine, USA). Rat cardiac troponin I ELISA kit (Cat#ab 266529) was purchased from Abcam (Cambridge, MA, USA). Rat intestinal fatty acid-binding protein (I-FABP/FABP2) kit (Cat#NBP2-82215) was purchased from Novus biologicals (Centennial CO, USA). Pierce chromogenic endotoxin quant kit (Cat#A39553) was purchased from Thermo Fisher Scientific (Rockford, IL, USA). Fatty acid-binding protein 1 (FABP-1)/liver fatty acid-binding protein (L-FABP) (Cat#DY 156505), TNFα (Cat#DY510), IL-1β (Cat#DY501), IL-4 (Cat# DY504), IL-10 (Cat# DY522) and IL-17F (Cat#DY4437) DuoSet ELISA kits were purchased from R&D systems (Minneapolis, MN, USA).

***TransRectal Intra-Colon (TRIC) temperature management catheter:*** TRIC device consists of: (i) a two-lumen catheter with an inflow tube, an outflow external membrane, (ii) temperature sensors and monitors, and (iii) a cooling fluid reservoir with a circulator pump (cooling system). Two temperature sensors (model HYP-3, CT, USA), were placed inside the ‘Inlet’ and ‘Outlet’ for recording the heat exchange between the colon-rectal segment of the catheter. ­­­

***Animal Preparation:*** Male Sprague Dawley rats weighing about 450 grams were purchased from Charles River (Boston, USA). Animals were kept under standardized conditions of temperature (22 ± 1°C), humidity (55% ± 5%), and 12:12 h light and dark cycles, and given ad libitum access to food and water. The bedding material is placed in each cage. Animal care staff and laboratory staff conduct routine husbandry procedures (e.g., cage cleaning, feeding, and watering) and check animals daily to assess their conditions. At one day before surgery, rats were assigned identification numbers and experimental groups, and then subjected to the pre-surgery behavior tests. Rats were fasted overnight (about 12 hours) by leaving 1.5 grams of food pellet in the cage but removing the rest of the food in the evening. On the day of surgery, the rat bodyweight, rectal temperature, surgical procedure, and anesthesia status were documented.

***Aseptic Survival Surgical Procedure:*** Rats were anesthetized with 4.5% isoflurane (VET ONE, Biose, USA) in a mixture of N2O:O2 (70:30) delivered to an induction chamber (Harvard Apparatus, Boston, USA) with scavenger (Harvard Apparatus, Boston, USA) attached for 5 minutes. Rats were then taken out and connected to a face mask for maintaining anesthesia with about 2% Isoflurane in a mixture of N2O:O2 (70:30) in a preparation bench area separated from the surgical table. Rats were administered with a subcutaneous injection of 0.02-0.05 mg/kg Buprenorphine (50-60 µl). Ocular protective lubricant (Optixcare Eye Lube, or Paralube, or Lacrilube, or Bauchlomb Soothe) was applied to both eyes and reapplied every 45-60 minutes until anesthesia was discontinued. Hair over the surgical sites was first clipped in a preparation area separated from the operating table. Nair hair remover lotion was then applied to the incision sites to remove remaining hair. The trans-rectal enema was performed with a syringe to remove contents in the descending colon.

Rats were then intubated with otoscope and a smooth-tip guide wire, moved to the operating table, and connected the endotracheal tube to a rat ventilator (Ugo Basile, Varesa, Italy, tidal volume 2.5 ~ 3.5 ml and ventilator rate is 65 breaths per minute). Anesthesia was maintained throughout surgery with 1.5 - 2% of isoflurane in a mixture of N2O:O2 (70:30) delivered via the endotracheal tube, connected to the ventilator. The depth of anesthesia was determined by lack of response to toe pinch and lack of palpebral reflex. The surgical skin areas were prepared with 3 alternating scrubs of betadine and 70% ethanol to the three surgical sites: chest, groin, and tail. The surgeon donned a disposable yellow gown, a facemask, washed hands with antiseptic soap solution, then donned sterile gloves. The assistant unpacked the first layer of sterile instruments, and the surgeon unpacked the second layer. A sterile surgical paper drape was used to cover the incision sites before placing a PE 50 catheter in the tail artery and the 2F Fogarty balloon REBOA catheter in the femoral artery. Another sterile surgical paper drape covered the upper body before placing a Silastic laboratory tubing (0.64 mm ID x 1.19 mm OD) in the right jugular vein for withdrawing blood and monitoring CVP, as well as placing a PE 20 catheter in the right brachial artery. Rats were administered with 30 U/kg heparin (100 U/ml, dilute 10 X) by the tail artery at 5 min before placing the REBOA balloon catheter.

***Rat Model of REBOA Management of Lethal Hemorrhage:*** The rat lethal exponential hemorrhage model was produced by withdrawing about 50% estimated blood volume (EVB) with a 20 ml syringe via the inferior vena cava catheter. The EBV was calculated using the following formula: EBV (ml) = 0.06 X bodyweight in kilogram + 0.77. Animals were bled 50% of EBV over 30 min in an exponential manner based on the method of Park et al. (4): 3.75 min = 10% EBV; 5.75 min = 10% EBV; 6.00 min = 10% EBV; 7.50 min = 10% EBV; and 8.00 min = 10% EBV hemorrhage. Small incisions in the neck, axilla, groin, and tail areas were made for inserting: (i) the inferior vena cava catheter line via the jugular vein in the neck for withdrawing blood; (ii) the right axillary arterial catheter for recording the proximal mean arterial pressure (MAP); (iii) the tail arterial catheter for recording the distal MAP; and (iv) a 2F Fogarty balloon catheter (Edwards Life Sciences, Irvine, USA) via the right femoral artery into the descending aorta at about 1 cm above the diaphragm for the implementation of REBOA. A laboratory-assembled TRIC temperature management catheter was inserted via the rectum into the descending colon at about 10 cm of length from the anus. A temperature sensor probe was inserted into the space between the colon inner surface and the surface of the TRIC catheter outer lumen to indicate the “intra-colon temperature”. A temperature sensor probe was embedded into the esophagus to indicate the upper body temperature, and into the bladder via the urinary tract to measure the bladder temperature for indicating the abdominal organ temperature. The intra-colon, intra-esophageal, and intra-bladder temperatures, as well as proximal and distal MAPs, were recorded throughout the experiment period with the Powerlab 16-channel data acquisition system (ADInstruments, Mountain View, CA, USA). The Arterial Blood Gas (ABG) (pH, pCO2, pO2, electrolytes, lactate, base deficit, bicarbonate, glucose, and potassium) were measured via an ABL90 FLEX blood gas analyzer (Radiometer, USA). The prothrombin time (PT)/international normalized ratio (INR) was measured via the Coag-Sense PT/INR Professional System according to manufacturer instructions (CoaguSense, Inc. Fremont, CA, USA).

After surgery, the rat was maintained for 10-20 min to establish stable baselines of temperatures (36-37ºC), as well as proximal and distal MAPs (80-110 mmHg). Hemorrhage was then introduced by continuously withdrawing blood via the inferior vena cava catheter over a period of 30 min as described above. The TRIC device was activated at 10 min after the blood withdrawal to induce either a rapid cooling of the colon inner surface to below 12ºC (TRIC12°C group) or maintaining the colon inner surface at 36-37ºC (TRIC37°C group). At the end of the hemorrhage period, the REBOA balloon was inflated to occlude the descending aorta at 1 cm above the diaphragm for 20 min. The esophageal, bladder, and the colonic wall inner surface temperatures were recorded (via the ADInstrument PowerLab). The proximal MAP was monitored via the right auxiliary artery catheter, while the distal MAP was via the tail artery catheter. Serial arterial blood samples were collected via the auxiliary artery catheter for the measurement of the blood gases at the following time points: (i) 2 min before hemorrhage, (ii) the end of hemorrhage but before REBOA (deployment), (iii) the end of REBOA, (iv) 15-30 min after deflation of REBOA balloon (post-REBOA), and (v) 60-90 min of post-REBOA.

After REBOA, the TRIC device remained active for 270 min during the post-REBOA temperature management period. In TRIC12°C group, rewarming was initiated at 180 min after REBOA with a rate of 0.15°C/min until the bladder temperature reached a range of 30°C, which took about an additional 90 min. Thus, the total TRIC temperature management period was 270 min.

At the end of temperature management, all catheters were removed, all incisions were closed, and isoflurane was switched off. A thin layer of lidocaine cream was layered on incisions. Once anesthesia was terminated, the rat was extubated when they were stable and breathing on their own. Buprenorphine (0.02-0.05 mg/kg SC, injection site prepared with 70% ethanol) was administered (6-12 hours after first administration). The rat was returned to the home cage. A Stoelting homoeothermic-heating pad (Cat# 50303, IL, USA) setting at 26-27°C was placed under the cage until they could ambulate on their own.

***Post-anesthesia care:*** During the first 3-6 h of recovery after the end of anesthesia, the rat was observed by experienced animal technicians and kept warm through the use of the Stoelting homoeothermic-heating pad under the cage until they ambulate on their own. The followings were monitored and documented daily during the post-anesthesia period: (i) motor and sensory functional score; (ii) body weight; and (iii) rectal temperature. At the experimental endpoint, rats were anesthetized with isoflurane and perfusion-fixed with formalin. Tissue samples of spinal cord (Lumbar L4-L5 segment), liver (left and median hepatic lobes), intestines (middle segments of small and large intestines), kidney (middle segments of left and right kidneys), and lung (middle segments of left and right lower lobes) were embedded into paraffin. Rats that were in moribund state (immediately before death; 3 of 5 rats) or dead for less than 3-4 hours (bodies were still warm and soft; 2 of 5 rats) before the endpoint were also fixed with formalin and the same areas of post-mortem organ samples were embedded into paraffin. Formalin fixed and paraffin embedded tissue sections (10 µm-thick) from the prescribed areas were stained with hematoxylin and eosin (H&E), and then examined with light microscopy in a double-blind fashion by two independent investigators. For the rats that died outside of the direct observation period and were included in the histopathological scoring, their brain and heart tissue and cell morphologies were checked to make sure that they were normal at the light microscopy and thus and the post-mortem deterioration was minimal. The following histopathological scoring criteria were used: 0 = normal tissue; 1 = selective cell necrosis without forming a necrotic area (more than 5 dead cell cluster), 2 = forming a small necrotic area (less than 10 necrotic cell cluster), 3 = more than 1 small necrotic areas or a large necrotic area (more than 10 necrotic cell cluster) without tissue destruction, and 4 = tissue destruction. If there was tissue hemorrhage, then 1 was added to the histological score. Sham-operated rats were subjected to the same surgical procedure, catheterization, TRIC12°C or TRIC37°C temperature managements, and post-anesthesia care but without implementations of hemorrhage and REBOA. One rat was excluded due to a vessel catheterization procedural accident and replaced with a new rat in the experimental group. These exclusion criteria were predefined in the original study design and not determined on a post-hoc basis.

***Euthanasia:*** Rats were euthanized under deep anesthesia (3-5% isoflurane) before collecting brain samples, which is consistent with the recommendations of the Panel on Euthanasia of the American Veterinary Medical Association.

***Blood sampling and plasma preparation:*** Blood samples were collected into heparinized tubes from the axillary artery catheter at pre-hemorrhage (0 min) and 90 and 180 minutes post-REBOA. Cells were removed from the plasma by centrifugation at 4°C, 1,600 g for 10 minutes. The resulting supernatant was immediately transferred into a clean propylene tube using a pipette via multiple aliquots and stored at -80°C until use.

***Measurement of plasma I-FABP/FABP2 and LPS:*** Intestinal injury was assessed by the plasma blood levels of fatty-acid binding protein (I-FABP), also known as FABP2, and endotoxin (also known as lipopolysaccharides) [16]. The plasma I-FABP and endotoxin concentrations were quantified with commercially available kits according to the manufacturer’s instructions (Novus Biologicals, CO, USA). The absorbance at 450 nm (for the FABP2 assay) and 405 nm (for the endotoxin assay) was recorded using a Varioskan Lux multimode microplate reader (Thermo Fisher Scientific, Waltham, Massachusetts, USA).

***Immunoassay for organ damage biomarkers:*** Liver injury was assessed by measuring the plasma levels of two key biomarkers, aspartate aminotransferase (AST) and fatty acid-binding protein 1 (FABP1). FABP1 is also known as liver-type fatty acid-binding protein (L-FABP). Kidney injury was assessed based on the elevation of plasma creatinine. An increase in creatinine is seen when there is approximately 50% loss of kidney function. Cardiac injury was evaluated by the elevation of plasma/serum cardiac troponin I (cTnI). The plasma levels of FABP1 (Novus Biologicals, CO, USA), creatinine (Sigma, St. Louis, MO, USA), and troponin I (Cambridge, MA, USA) were determined by commercially available kits according to the manufacturer’s instructions. The absorbance at 450 nm (for AST and cTnI) and 570 nm (for creatinine) was recorded using a Varioskan lux multimode microplate reader. A wavelength correction was applied to determine the concentration of FABP-1 by subtracting the absorbance at 570 nm from 450 nm.

**Cytokine Assay:** The plasma levels of pro-inflammatory cytokines (TNFα, IL-1β, and IL-17F) and anti-inflammatory cytokines (IL-4 and IL-10) were determined by using ELISA kits according to manufacturers’ instructions (R&D Systems, MN, USA). The plasma samples were diluted 1:10 with the diluent reagent in the kits before each assay. For optical density determination, wavelength correction was applied by subtracting the reading at 570 nm from the reading at 450 nm using the Varioskan lux multimode microplate reader.

***Statistical Analysis:*** Data are expressed as mean ± standard error of the mean (SEM). One-way ANOVA followed by Tukey post-hoc test for comparison of more than 2 experimental groups for statistical analysis of values of tissue injury biomarkers and cytokines. The log-rank test for survival rate analysis. Chi-Square test for mortality rate. GraphPad Prism version 7.00 for Windows was used (GraphPad Software, La Jolla, California, USA). p<0.05 was considered a statistically significant difference.

| **Supplemental Table 1. Intra-Colon Temperature** | | | | | | | |
| --- | --- | --- | --- | --- | --- | --- | --- |
| **Exp. Group** | **Pre-H** | **End-H** | **REBOA** | | | **Post-REBOA** | |
|  |  |  | **0-5'** | **5'-15'** | **15'-30'** | **0-5'** | **1h** |
| **25’ REBOA + TRIC 37°C** | 36.99 ± 0.34 | 36.45 ± 0.56 | 36.25 ± 0.72 | 35.74 ± 2.12 | 35.48 ± 1.15 | 35.85 ± 0.76 | 36.87 ± 0.06 |
| **25’ REBOA + TRIC 12°C** | 36.89 ± 0.28 | 9.63 ± 1.74 | 9.27 ± 1.72 | 9.69 ± 1.04 | 10.41 ± 2.05 | 10.90 ± 2.51 | 10.99 ± 1.54 |
| **30’ REBOA + TRIC 12°C** | 36.79 ± 0.31 | 8.65 ± 1.23 | 8.41 ± 1.12 | 9.32 ± 1.25 | 10.40 ± 1.35 | 10.56 ± 1.14 | 10.42 ± 1.12 |
| **30’ REBOA + Hex + TRIC 12°C** | 36.77 ± 0.31 | 8.91 ± 1.28 | 8.11 ± 1.13 | 9.58 ± 1.13 | 10.46 ± 1.14 | 10.58 ± 1.13 | 10.58 ± 1.13 |

**Supplemental Table 1.** Intra-colon temperature throughout the 270 min of TRIC temperature management period. Experimental (Exp.) groups are: (i) 25 min REBOA + TRIC37°C; (ii) 25 min REBOA + TRIC12°C; (iii) 30 min REBOA + TRIC12°C; and (iv) 30 min REBOA + TRIC12°C + 0°C HEX. Data are mean ± SEM (n=3 for each groups). Pre-H = Pre-hemorrhage, i.e., at the time point of 5 min before induction of hemorrhage; End-H = End-hemorrhage, i.e., at the end of blood withdrawal (for 30 min); REBOA = Resuscitative Endovascular Balloon Occlusion of the Aorta; Post-REBOA = after deflation of the REBOA balloon.

| **Supplemental Table 2. Esophageal Temperature** | | | | | | | |
| --- | --- | --- | --- | --- | --- | --- | --- |
| **Exp. Group** | **Pre-H** | **End-H** | **REBOA** | | | **Post-REBOA** | |
|  |  |  | **0-5'** | **5'-15'** | **15'-30'** | **0-5'** | **1h** |
| **25’ REBOA + TRIC 37°C** | 37.14 ± 0.04 | 36.48 ± 0.41 | 36.21 ± 0.36 | 36.11 ± 0.04 | 36.25 ± 0.47 | 36.98 ± 0.16 | 36.92 ± 0.16 |
| **25’ REBOA + TRIC 12°C** | 36.99 ± 0.30 | 34.95 ± 0.21 | 34.04 ± 0.31 | 32.57 ± 0.52 | 31.10 ± 0.61 | 25.90 ± 0.61 | 25.10 ± 0.61 |
| **30’ REBOA + TRIC 12°C** | 36.89±0.14 | 34.65±0.48 | 33.84±0.42 | 32.19±0.55 | 30.44±0.74 | 29.10±0.78 | 25.33 ± 0.66 |
| **30’ REBOA + Hex + TRIC 12°C** | 36.87±0.08 | 34.89±0.31 | 33.83±0.33 | 32.16±0.36 | 29.98±0.78 | 27.06±0.64 | 25.12 ± 0.45 |

**Supplemental Table 2.** Esophageal temperature throughout the 270 min of TRIC temperature management period. Experimental (Exp.) groups are the same as in Supplemental Table 1.

| **Supplemental Table 3. Proximal or Upper Body MAP** | | | | | | | |
| --- | --- | --- | --- | --- | --- | --- | --- |
| **Exp. Group** | **Pre-H** | **End-H** | **REBOA** | | | **Post-REBOA** | |
|  |  |  | **0-5'** | **5'-15'** | **15'-30'** | **0-5'** | **1h** |
| **25’ REBOA + TRIC 37°C** | 88.99 ± 4.48 | 27.86 ± 1.76 | 104.5 ± 3.97 | 120.89 ± 0.12 | 123.9 ± 1.66 | 75.08 ± 1.08 | 74.32 ± 4.75 |
| **25’ REBOA + TRIC 12°C** | 89.95 ± 4.48 | 25.86 ± 2.31 | 92.32 ± 3.97 | 122.08 ± 3.55 | 121.1 ± 1.37 | 85.60 ± 3.59 | 75.31 ± 8.86 |
| **30’ REBOA + TRIC 12°C** | 91.03 ± 5.55 | 26.86 ± 1.58 | 114.8 ± 7.88 | 135.08 ± 3.99 | 125.8 ± 4.35 | 83.35 ± 2.37 | 66.94 ± 4.31 |
| **30’ REBOA + Hex + TRIC 12°C** | 90.40 ± 6.51 | 27.03 ± 2.17 | 131.5 ± 4.43 | 145.18 ± 5.84 | 147.7 ± 5.08 | 94.91 ± 13.31 | 76.94 ± 3.49 |

**Supplemental Table 3.** The proximal or upper body mean arterial blood pressure (MAP) was recorded via the right axillary artery catheter. Experimental (Exp.) groups are the same as in Supplemental Table 1.

| **Supplemental Table 4. Distal or Lower Body MAP** | | | | | | | |
| --- | --- | --- | --- | --- | --- | --- | --- |
| **Exp. Group** | **Pre-H** | **End-H** | **REBOA** | | | **Post-REBOA** | |
|  |  |  | **0-5'** | **5'-15'** | **15'-30'** | **0-5'** | **1h** |
| **25’ REBOA + TRIC 37°C** | 88.02 ± 3.72 | 22.66 ± 3.14 | 12.84 ± 0.57 | 16.61 ± 0.61 | 17.30 ± 1.03 | 63.92 ± 7.27 | 70.66 ± 3.18 |
| **25’ REBOA + TRIC 12°C** | 83.37 ± 4.67 | 21.86 ± 2.44 | 14.20 ± 1.96 | 15.72 ± 2.16 | 18.15 ± 1.58 | 78.66 ± 5.59 | 83.26 ± 9.26 |
| **30’ REBOA + TRIC 12°C** | 87.19 ± 6.64 | 21.02 ± 0.61 | 11.26 ± 0.69 | 14.33 ± 0.29 | 16.29 ± 1.15 | 57.50 ± 3.08 | 56.55 ± 7.20 |
| **30’ REBOA + Hex + TRIC 12°C** | 89.38 ± 5.71 | 23.67 ± 1.13 | 12.79 ± 2.43 | 17.95 ± 2.14 | 21.15 ± 3.87 | 67.91 ± 13.44 | 76.59 ± 1.41 |

**Supplement Table.4.** The distal or lower body MAP was recorded via the tail artery catheter. Experimental (Exp.) groups are the same as in Supplemental Table 1.

| **Supplemental Table 5. Heart Rate (bpm)** | | | | | | | |
| --- | --- | --- | --- | --- | --- | --- | --- |
| **Exp. Group** | **Pre-H** | **End-H** | **REBOA** | | | **Post-REBOA** | |
|  |  |  | **0-5'** | **5'-15'** | **15'-30'** | **0-5'** | **1h** |
| **25’ REBOA + TRIC 37°C** | 396 ± 0.00 | 380 ± 12.14 | 344 ± 14.97 | 332 ± 14.64 | 340 ± 18.57 | 388 ± 31.57 | 420 ± 12.14 |
| **25’ REBOA + TRIC 12°C** | 384 ± 10.19 | 378 ± 11.70 | 312 ± 14.00 | 296 ± 11.65 | 248 ± 16.81 | 228 ± 13.81 | 216 ± 14.99 |
| **30’ REBOA + TRIC 12°C** | 388 ± 12.97 | 376 ± 18.49 | 324 ± 10.78 | 300 ± 10.78 | 252 ± 17.71 | 248 ± 15.43 | 232 ± 18.49 |
| **30’ REBOA + Hex + TRIC 12°C** | 386 ± 13.85 | 376 ± 12.00 | 297 ± 15.98 | 273 ± 13.07 | 248 ± 18.33 | 200 ± 18.33 | 136 ± 13.85 |

**Supplemental Table 5.** Heart rate (beats per minute or bpm) was recorded via electrocardiogram (ECG). Experimental (Exp.) groups are the same as in Supplemental Table 1.


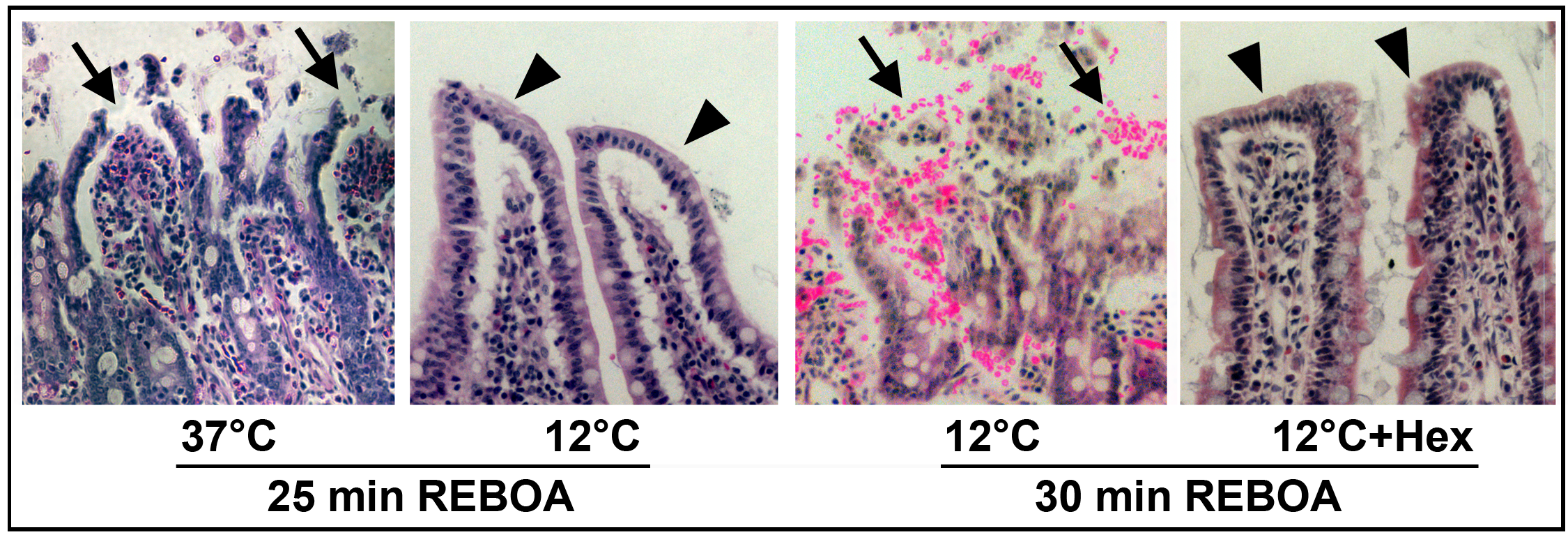


Supplemental Fig. 1. Light micrographs of H&E-stained intestine (ilium) tissue sections, Experimental groups were: (i) 25 min REBOA + TRIC37°C; (ii) 25 min REBOA + TRIC12°C; (iii) 30 min REBOA + TRIC12°C; and (iv) 30 min REBOA + TRIC12°C + 0°C Hex. Arrows indicate mucosal damage and villi destruction. Arrowheads point to relatively intact mucosa.
